# Screening for large rearrangements of the *RB1* gene in Iranian patients with retinoblastoma using multiplex ligation-dependent probe amplification

**Published:** 2013-02-22

**Authors:** Ali Ahani, Mohammad Taghi Akbari, Kioomars Saliminejad, Babak Behnam, Mohammad Mehdi Akhondi, Parvaneh Vosoogh, Farriba Ghassemi, Masood Naseripour, Gholamreza Bahoush, Hamid Reza Khorram Khorshid

**Affiliations:** 1 Reproductive Biotechnology Research Center, Avicenna Research Institute, ACECR, Tehran, Iran; 2 Department of Medical Genetics, Faculty of Medical Sciences, Tarbiat Modares University, Tehran, Iran; 3 Department of Genetics and Molecular Biology, Tehran University of Medical Sciences, Tehran, Iran; 4 Pediatric Oncology Center, Mahak Hospital, Tehran, Iran; 5 Eye Research Center, Farabi Hospital, Tehran University of Medical Sciences; 6 Eye Research Center, Rasoul Akram Hospital, Tehran University of Medical Sciences; 7 Oncopathology Research Center, Ali-Asghar Children Hospital, Tehran University of Medical Sciences; 8 Genetic Research Center, University of Social Welfare and Rehabilitation Sciences, Tehran, Iran

## Abstract

**Purpose:**

To screen deletions/duplications of the *RB1* gene in a large cohort of Iranian patients using the multiplex ligation-dependent probe amplification (MLPA) technique.

**Methods:**

A total of 121 patients with retinoblastoma, involving 55 unilateral and 66 bilateral or familial retinoblastomas, were included in this study. Among these patients, 121 blood and 43 tissue samples were available. DNA was extracted from the blood and tissue samples and analyzed with an *RB1-*specific MLPA probe set. The mutation findings were validated with SYBR Green Real-Time PCR.

**Results:**

Twenty-two mutations were found in 21 patients; of these, ten mutations were detected in patients with isolated unilateral retinoblastoma.

**Conclusions:**

Our results suggested that MLPA is a fast, reliable, and powerful method for detecting deletions/duplications in patients with retinoblastoma.

## Introduction

Retinoblastoma, with an estimated frequency of 1:15,000, is the most common intraocular solid tumor in children under 6 years of age [1]. Inactivation of both alleles of the *RB1* gene in a single immature retinal cell can trigger retinoblastoma tumorigenesis. In about 50% of patients, both inactivating mutations occur somatically, whereas in the hereditary form, a mutated allele is inherited, and the second mutational event occurs somatically. The latter group often presents with the disease at an earlier age in both eyes [2,3].

The large *RB1* gene spans more than 180 kb on chromosome 13q14, which consists of 27 exons and transcribes into 4.8 kb messenger RNA [4]. Thus far, wide spectrums of the mutations including large cytogenetic rearrangements, subcytogenetic deletions/duplications, and point mutations have been reported in the *RB1* gene. With the exception of recurrent mutations in 11 arginine codons, the *RB1* gene has no remarkable mutation hot spots and point mutations, which are usually unique to each family, distributed all over it [4-12].

Detecting *RB1* mutations could enhance the quality of clinical management of retinoblastoma in patients, and risk prediction for all members of affected families could be estimated [13]. Despite these advantages, there are many challenges in molecular genetic diagnosis of retinoblastoma because of the large size of the *RB1* gene and widely dispersed mutations [13,14].

Currently, the routine procedure for *RB1* gene testing is joint screening of the coding regions with direct sequencing and deletions/duplications analysis. Fluorescent in situ hybridization (FISH) and karyotyping were previously used to evaluate cytogenetic abnormalities but have been replaced by more enhanced and specialized techniques such as multiplex ligation-dependent probe amplification (MLPA) or quantitative multiplex fluorescence PCR (QMFPCR). Together, these approaches could detect more than 90% of all known mutations [11,15].

In the past decade, MLPA has been accepted as a sensitive method for detecting cytogenetic and subcytogenetic abnormalities and has been merged in the gene testing procedure in many laboratories [16]; however, there is little information about the ability of this technique in the *RB1* gene literature [11,17,18]. Accordingly, in the present study, the detection rate of *RB1* gene gross rearrangements in a large cohort of Iranian patients with retinoblastoma was investigated with MLPA.

## Methods

### Subjects

A total of 121 patients with retinoblastoma (55 patients with isolated unilateral retinoblastoma, one patient with hereditary unilateral retinoblastoma, 53 patients with isolated bilateral retinoblastoma, and 12 patients with hereditary bilateral retinoblastoma) who were referred to Mahak, Farabi, and Rasoul Akram hospitals were included in this study. The mean ages of the patients with unilateral and bilateral retinoblastoma were 21.7 and 14.4 months, respectively. Among these, enucleated eye samples from 43 patients were also recruited for molecular analysis. In the patients with unilateral retinoblastoma, 23 out of 56 were enucleated, and tumor samples were available; only one patient had a family history of retinoblastoma. In the bilateral group, 20 patients were enucleated, and 12 patients had a family history of retinoblastoma.

From each patient, 5 ml of peripheral blood in tubes containing EDTA and 2 ml in heparin tubes were collected. Genomic DNA from peripheral blood samples was extracted using standard salting out method. DNA from the tumor samples were extracted by heat-induced retrieval protocol (boiling of tissue sections in 0.1 M alkaline solution) [19]. All patients’ families were subjected to genetic counseling, and informed consent was obtained from each parent/guardian. The study protocol was approved by the Avicenna Research Institute's Ethics and Human Rights Committee. The study was in accordance with the provisions of the Declaration of Helsinki. Clinically, retinoblastoma was diagnosed by the presence of tumors in one or both eyes, and diagnosis for enucleated patients was confirmed with pathological analysis.

### Multiplex ligation-dependent probe amplification

To investigate large deletions/duplications in the *RB1* gene, MLPA analysis was performed using the SALSA MLPA kit P047-B1 RB1 (MRC-Holland, Amsterdam, the Netherlands) according to the manufacturer’s protocol. The kit contained 24 probes for the *RB1* gene (the promoter and each exon had a specific probe, except exons 5, 10, 15, and 16), three probes for the flanking genes of the *RB1* gene, *ITM2B*, *CHC1L*, and *DLEU1*, and 13 control probes on locations other than chromosome 13. *CHC1L* and *DLEU1* are centromeric whereas *ITM2B* is telomeric to RB1. The average distances between *RB1* and *ITM2B, CHC1L,* and *DLEU1* are 50 kb, 50 kb, and 1.5 Mb, respectively.

Briefly, 100 ng of genomic DNA in a final volume of 5 µl was denatured and hybridized with the SALSA probe mix, followed by incubation at 60 °C for 18 h. Subsequently, the annealed probes were ligated using the Ligase-65 mix provided at 54 °C for 15 min.

In the next step, 10 µl of ligated products, as the template, were used for DNA amplification. The PCR amplicons were run on a Genetic Analyzer 3130 (Applied Biosystems, Foster City, CA), and the results were analyzed with GeneMarker software version 1.91 (SoftGenetics LLC, State College, PA). The normal pattern was expected to produce a normalized signal value ratio of 1:1; any value out of the ranges <0.75 or >1.30 was considered abnormal and corresponded to a deletion and a duplication, respectively.

### Inclusion criteria for control samples

In each MLPA reaction, regarding the number of samples, three to six control samples were simultaneously used. All controls were adults, with no ocular tumor or other malignancy. In addition, the locations of all internal probes of the *RB1* gene in the control group were verified with direct sequencing. To confirm the presence of two normal copies of the *RB1* gene and the absence of any chromosomal deletions, all control samples were checked with the two STS markers, D13S153 (Rbi2, inside *RB1* intron 2) and D13S128 (in the flanking sequence of the *RB1* gene). Only samples heterozygous for both markers were included.

### Validation of multiplex ligation-dependent probe amplification results

Samples with abnormal MLPA results were checked with direct sequencing to be assured of an intact probe binding site and appropriate binding of MLPA probes to their related regions; the primers and PCR conditions were described previously [11]. Gene dosage for different samples was performed with relative quantification, and 2^-ΔΔCt^ was calculated by normalizing the *RB1* exons to the *RPPH1* gene, a single copy reference gene. In addition, fragments with similar size and GC content to *RPPH1* were designed for the *RB1* exons in which deletions and duplications were observed with MLPA. Only those with similar efficiency to *RPPH1* were used to evaluate the gene dosage. The primer sequences and sizes are shown in Table 1. Real-time PCR (SYBR Green) was performed using a serial dilution of DNA samples including 200, 100, 50, 25, 12.5, 6.25, and 3.125 ng in quadruple repeats. Then, according to the standard curves created and by comparing the slope and efficiency of each reaction, 25 ng of DNA was chosen as the best concentration that gave dose-dependent results. In this DNA concentration, real-time PCR detected samples with deletion or duplication. The copy numbers of the exons compared to the reference gene were determined as follows: ΔΔCt=(Ct *RPPH1* (calibrator sample) − Ct *RB1* exon (calibrator sample)) – (Ct *RPPH1* (unknown sample) − Ct *RB1* exon (unknown sample)). Absolute quantification was made by converting the measured values to absolute ones. Then using the ratio equation (2^−ΔΔCt^), the relative gene copy numbers were calculated. The expected values were about 1 for normal samples, 0.5 for heterozygous deletions, and 1.5 for heterozygous duplications [20].

**Table 1:** The primers used for quantitative analysis of *RB1* gene.

Genomic region	Primers (5′ → 3′)		Size (bp)	Ta
*RPPH1*	Forward	GAGGTGAGTTCCCAGAGAACG	134	60
	Reverse	TTCGCTGGCCGTGAGTCTGTTC		
*RB1* exon 7	Forward	TCAGGGGAAGTATTACAAATGGAAG	117	60
	Reverse	ACTATATGGTTCTTTGAGCAACATG		
*RB1* exon 13	Forward	CTAAAGCTGTGGGACAGGGTTG	116	60
	Reverse	TTATACGAACTGGAAAGATGCTGC		
*RB1* exon 17	Forward	GCCTTTGATTTTTACAAAGTGATCGAAAG	128	60
	Reverse	CTTACTGAGAGCCATGCAAGGGA		
*RB1* exon 19	Forward	TATATCTAGGTATCTTTCTCCTGTAAG	130	60
	Reverse	GGTAGATTTCAATGGCTTCTGGG		
*RB1* exon 22	Forward	TTGCAGTATGCTTCCACCAGG	123	60
	Reverse	GGTAGGGGGCTAGAGCAAAAAC		

### Karyotype analysis

To investigate cytogenetic abnormalities in the patients, fresh blood samples were taken in heparin tubes, cultured on RPMI-1640 medium, and finally GTG banded according to standard protocols. The prepared slides were directly analyzed under the microscope to evaluate any potential chromosomal changes. Each slide had approximately 440 to 500 bands.

## Results

**Figure 1:**
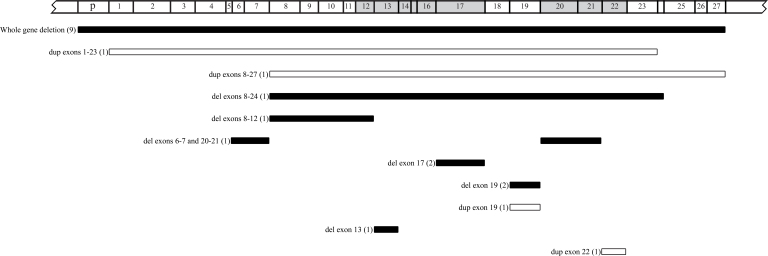
Schematic representation of the deletions and duplications found in this study. Arabic numbers in the parentheses show the occurrence times for each rearrangement. The white bars represent duplications, and the black ones indicate deletions. The gray regions on the *RB1* gene show the pocket domains.

Cytogenetic analysis for all patients showed a normal 46, XY or 46, XX karyotype. MLPA reactions were performed for all 121 samples, resulting in 22 mutations identified in 21 patients; among these mutations, nine whole gene deletions, nine intragenic deletions, and four intragenic duplications were observed (Table 2 and Figure 1). In eight of the nine patients with whole gene deletion, all three flanking probes signals showed a deletion, which indicated a deletion of at least 1.5 Mb; however, in one bilateral patient, only the two probes for *ITM2B* and *CHC1L* were deleted. All mutations identified with MLPA were validated using real-time PCR analysis; the sequencing results also showed that there is no point mutation in the probe-binding site of the samples.

**Table 2:** The rearrangements found in the 21 retinoblastoma patients detected by MLPA in tumor as well as blood samples.

**Patient’s ID**	**Mutation in tumor sample**	**Mutation in blood sample**	**Type of disease**	**Familial History**
IRB1	Del Ex 8 −12	Normal	Unilateral	No
IRB12	Whole gene deletion	Whole gene deletion	Unilateral	No
IRB13	Del Ex 17	Normal	Unilateral	No
IRB14	Del Ex 8–24	Del Ex 8–24	Bilateral	No
IRB15	Del Ex 6–7, Del Ex 20–21	Del Ex 6–7	Bilateral	No
IRB19	Dup Ex 19	Dup Ex 19	Unilateral	No
IRB22	Dup Ex 8–27	Normal	Unilateral	No
IRB28	Whole gene deletion	Normal	Unilateral	No
IRB35	Dup Ex 1–23	Normal	Unilateral	No
IRB38	Whole gene deletion	Whole gene deletion	Bilateral	No
IRB40	Dup Ex 22	Dup Ex 22	Bilateral	No
IRB41	Whole gene deletion	Whole gene deletion	Bilateral	No
IRB113	No tumor sample available	Del Ex 13	Unilateral	No
IRB120	No tumor sample available	Whole gene deletion	Bilateral	No
IRB133	No tumor sample available	Whole gene deletion	Bilateral	No
IRB139	No tumor sample available	Whole gene deletion	Bilateral	No
IRB143	No tumor sample available	Whole gene deletion	Unilateral	Yes
IRB158	No tumor sample available	Whole gene deletion	Unilateral	No
IRB159	No tumor sample available	Del Ex 19	Bilateral	No
IRB179	No tumor sample available	Del Ex 19	Unilateral	No
IRB194	No tumor sample available	Del Ex 17	Bilateral	No

Del: Deletion; Ex: Exon; Dup: Duplication.

**Figure 2:**
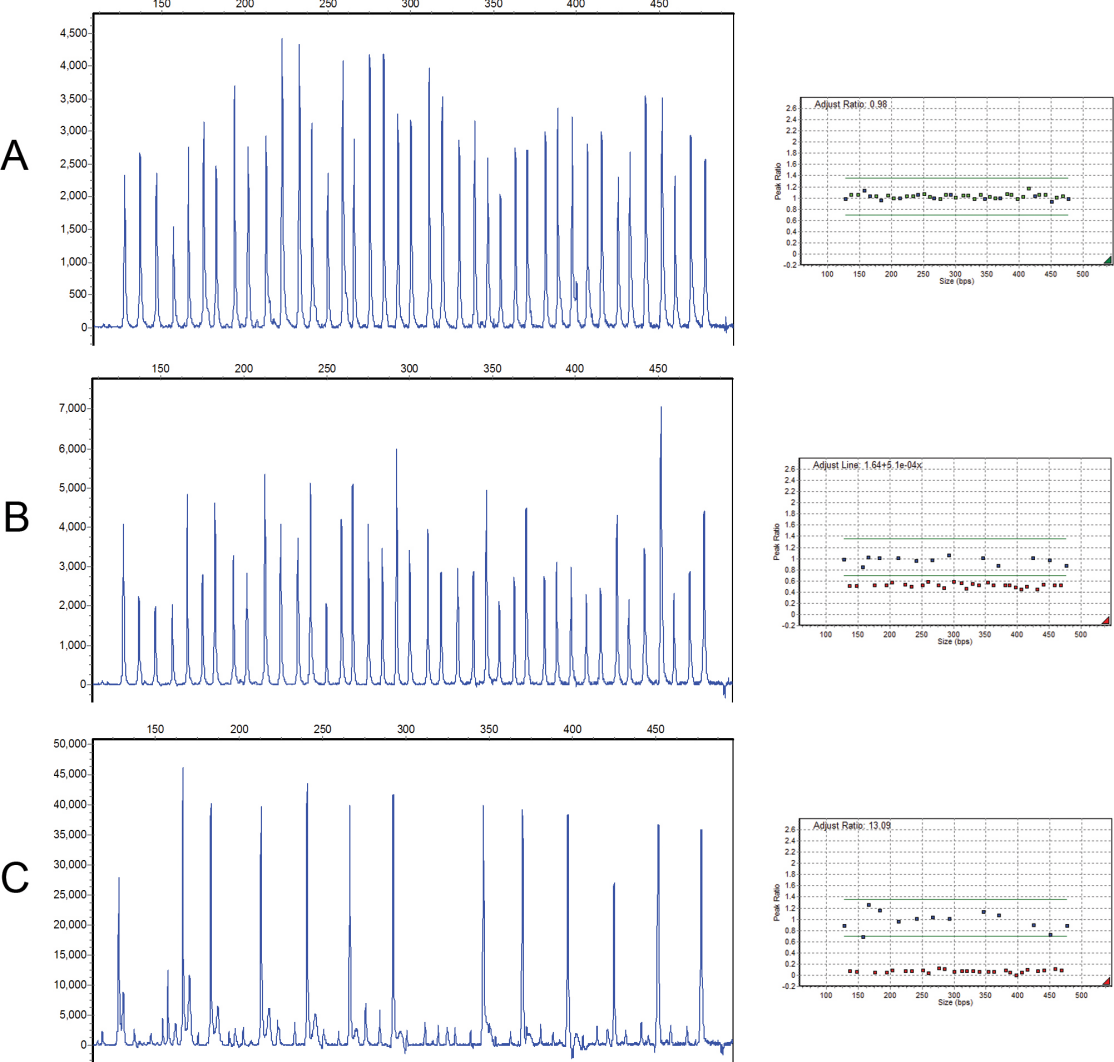
Chromatograms illustrating whole gene deletion in patient IRB12. **A**: Normal control. **B**: Multiplex ligation-dependent probe amplification (MLPA) results of a blood DNA sample shows heterozygous deletion of the *RB1* gene. **C**: The later MLPA results for the tumor DNA sample of the same patient show homozygous deletion of *RB1*.

**Figure 3:**
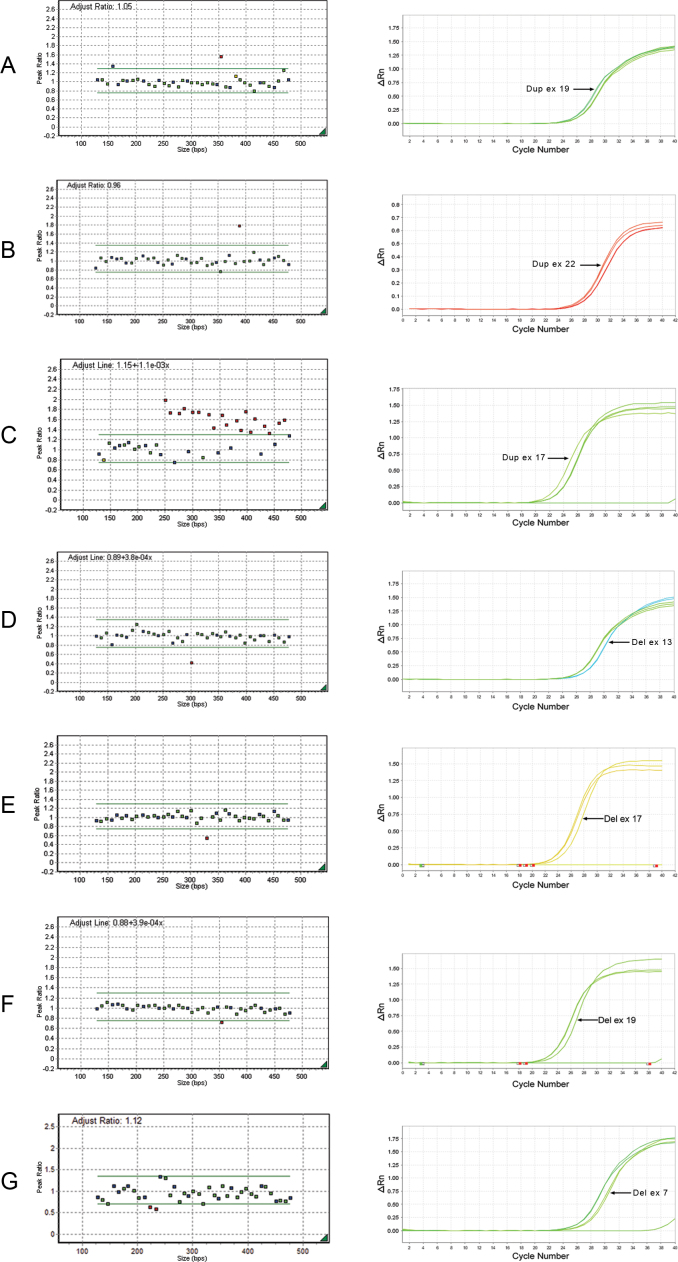
GeneMarker plots of multiplex ligation-dependent probe amplification reactions (left) and corresponding real-time polymerase chain reactions (right) to validate the results of (**A**) exon 19 duplication, (**B**) exon 22 duplication, (**C**) exon 8 through 22 duplication, (**D**) exon 13 deletion, (**E**) exon 17 deletion, (**F**) exon 19 deletion, and (**G**) exon 6 and 7 deletion.

Regarding disease type, ten large mutations were found in patients with unilateral retinoblastoma (ten of 55); five of these patients showed abnormalities in either blood and tumor samples. According to these results, the MLPA detection rate for constitutional mutations in patients with unilateral retinoblastoma (five of 55) was 9.1%. The total detection rate for unilateral cases (10 of 55) was 18.2% and for bilateral and familial cases was 11 of 66 (16.6%). Sixteen mutations in 121 (13.2%) blood samples were detected with MLPA. The MLPA results are shown in Figure 2 and Figure 3. Examples of standard curves and amplification plots of real-time PCR demonstrating deletions in two patients are elucidated in Figure 3.

## Discussion

MLPA as a sensitive, reproducible, and sequence-specific technique was described by Schouten et al. for detecting gains or losses of single exons in small amounts of human DNA samples [16]. Now MLPA is a reliable method for detecting large deletions/duplications. Despite the growing number of studies that have used MLPA to analyze genes in various human diseases [21-25], only a few have used it in evaluating *RB1* mutations in retinoblastoma and other cancers [17,18,26-28]. Although many laboratories use MLPA for detecting and screening large *RB1* mutations, there is no comprehensive data in the literature. In this survey, 121 patients with retinoblastoma were evaluated with MLPA.

Cytogenetic abnormalities and subcytogenetic mutations cause many retinoblastomas [29-32]. Approximately 15%–25% of retinoblastoma cases are due to large deletions and insertions [11,13,14]. These rearrangements were previously investigated with various techniques such as karyotyping, G-banding, FISH, QMFPCR, MLPA, and real-time PCR [5,10,14,33-35]. However, each technique has its own advantages and limitations. Karyotyping and FISH can detect only large rearrangements including entire gene deletion. These techniques are especially useful in determining the borders of large deletions that have been identified by other methods. Other methods such as MLPA, real-time PCR, and QMFPCR, which are based on quantification of amplifying PCR products, are more sensitive and can detect more mutations. Real-time PCR assays are characterized by high precision; however, they are difficult to implement as a multiplex. MLPA and QMFPCR can be easily multiplexed. Hence, in a single reaction, all regions of a gene can be analyzed, and an abnormality is found, it can reanalyzed with real-time PCR. QMFPCR should be set up manually, which makes the technique error-prone and reduces its reproducibility, whereas MLPA probes and reagents are commercially available and easy to use. Despite the ability of MLPA and QMFPCR to identifying the numerical changes in DNA, the techniques cannot detect mutations such as translocations and inversions, while karyotyping can detect this type of aberration.

In the present study, no *RB1* mutations including large deletion, translocation, and inversion were detected with karyotyping. By analyzing the MLPA results, 22 deletions/duplications in 21 patients were found. Among these mutations, 16 were detected in the blood and tumor samples. The frequency of deletions/duplications in previous studies varies from 10% to 20% [5,7,13,14]. In our study, the frequency of constitutional deletions/duplications in isolated unilateral and bilateral/familial tumors were 9.1% (five of 55) and 16.6% (11 of 66), respectively. According to our results, 17.3% (21 of 121) of the total mutations were detected with MLPA, a rate similar to previous studies. Germline mutations in 10%–13% of patients with unilateral retinoblastoma have been reported [4,13,14,36-38]. Our estimation of the deletions/duplications rate using MLPA was 9.1% for patients with isolated unilateral retinoblastoma. Comparison of data shows that the MLPA detection rate in these patients was nearly equal to the total mutations (including deletion/duplication and point mutations) found in the other studies. However, our frequency of deletions/duplications in patients with unilateral retinoblastoma is higher than previous reports. This could be explained by two hypotheses: First, the small size of the population (55 samples) could result in chance findings. Second, the higher detection rate could be due to the higher sensitivity of MLPA compared to the methods used in other studies.

Analysis of larger sample sizes may show that this high rate of alterations is a chance finding. However, according to the results, MLPA should be considered for unilateral retinoblastoma. Therefore, to find causal mutations, if tumor samples are not available, MLPA could be recommended as the first step of mutation detection. However, if no mutation is detected with MLPA, other popular methods including sequencing of the entire *RB1* coding region is suggested.

Recently, Rushlow et al. [15] showed that a remarkable number of patients with retinoblastoma carry mosaic mutations; the researchers found mutations in 92.6% of cases via a combination of full sequencing and deletions/duplication analysis of *RB1*. Moreover, they found additional mutations in cases with clearly normal results using PCR-based methods, so the detection rate increased to 94.8% [15]. Results of MLPA or real-time PCR in low-level mosaic cases, depending on the percentage of mutant cells, may be mistaken as normal. Actually, MLPA is a relative quantification method, and deletions/duplications in unknown samples are identified by comparison to the normal controls. MLPA is not expected to detect all imbalances in mosaic cases [39].

Germline mutations in unilateral cases such as splice site affecting and missense mutations out-of-pocket domains usually have mild to moderate deleterious effects [40,41]. In this regard, based on our findings, deletions/duplications have a relatively weak to moderate effect as well. There is considerable evidence of the genetic mechanisms that explain the incomplete penetrance of single exonic deletions (for example, exon 4) or multiexonic large deletions such as exons 24–25 [42-44]. In such mutations, the in-frame exonic deletions cause the pRB to lose some of its function as well as penetrance of the mutation, which depends on the remaining activity of the shortened protein. In addition, as previously described, the whole gene deletions show incomplete penetrance as well [5,45-47]. However, when age is addressed, the unilateral tumors may be due to the patients’ young age. Patients with unilateral retinoblastoma, as a progressive consequence of the disease, may probably show bilateral tumors in the future. Both unilaterally affected children (3 and 8 months) in our study with whole gene deletion were treated with systemic chemotherapy, which may prevent formation of new tumors in the other eye. Thus, these patients may be incorrectly categorized in the unilateral group. To clarify this, further long-standing investigation and follow-up are necessary.

In conclusion, MLPA is a strong method for primary evaluation of *RB1* gene deletions/duplications in patients with retinoblastoma. Therefore, MLPA is recommended as a fast method in primary screening of retinoblastoma.
